# Death of Dr. Lockwood

**Published:** 1871-07

**Authors:** 


### Death of Dr. Lockwood.

A special meeting of the Erie County Medical Society was held at their rooms, in the Young Men's Association building, at 12½ P. M, on Tuesday, Dec., 27th, 1870, the object of the meeting being to pay a proper tribute of respect to the memory of their late associate, Dr. T. T. Lockwood.

After appropriate remarks by several members of the Society, the Committee on resolutions reported the following, which were unanimously adopted :

*Resolved*, That in the death of Dr. Timothy T. Lockwood, we mourn the loss of one of our oldest and most highly respected members, who was especially endeared to us by his unswerving fidelity to the interests and honor of the profession, by his earnest discharge of all the duties and obligations of professional life, and by his constancy and faithfulness as a friend, in all these respects, leaving us an example worthy of imitation.

*Resolved*, That we will attend his funeral in a body and wear the usual badge of mourning.

*Resolved*, That a copy of these proceedings be transmitted to the family of the deceased, and that the same be published in the Buffalo *Medical and Surgical Journal* and in the daily papers of the city.

J. B. SAMO, *Secretary.*

